# Application of tobacco control experience to alcohol consumption measures

**DOI:** 10.2471/BLT.25.294093

**Published:** 2026-04-16

**Authors:** Prashant Kumar Singh, Chandan Kumar, Shalini Singh

**Affiliations:** a Division of Preventive Oncology and Population Health, WHO Framework Convention on Tobacco Control Knowledge Hub on Smokeless Tobacco, ICMR National Institute of Cancer Prevention and Research, Indian Council of Medical Research, I-7 Sector-39, Noida, Uttar Pradesh, 201301, India.; b Department of Policy and Management Studies, TERI School of Advanced Studies, New Delhi, India.

## Abstract

Low- and middle-income countries face a rapidly escalating, alcohol-related public health crisis spanning cancer, injury, liver disease, mental health disorders and the complications of human immunodeficiency virus infections. Despite compelling evidence that alcohol is a leading risk factor for premature death and disability, few countries include comprehensive alcohol control policies in their public health agenda. This gap between evidence and policy is critical because evidence-based interventions could decrease the alcohol-attributable disease burden by up to 30% within a decade. We conducted a policy analysis to synthesize evidence from low- and middle-income countries and propose a comprehensive multicomponent framework for alcohol control adapted from experience with tobacco control. Documented achievements show that simultaneously integrating alcohol control into the existing health-care system, introducing regulation, and building institutional capacity can reduce consumption and generate substantial economic returns. However, interference from the alcohol industry in policy, mirroring the historical approach of the tobacco industry, underscores the need for explicit protections. Evidence-based recommendations on implementing alcohol control policies are presented for international agencies, national governments, health-care systems and civil society. These recommendations also draw on global lessons from the World Health Organization’s Framework Convention on Tobacco Control. Today, the convergence of robust scientific evidence, proven interventions, successful implementation models and growing political support present an opportunity to advance alcohol control in low- and middle-income countries. The question is not whether comprehensive alcohol control is feasible in resource-constrained settings but whether the global health community will act decisively on the evidence available.

## Résumé

Les pays à revenu faible et intermédiaire sont confrontés à une crise de santé publique liée à l’alcool qui s’aggrave rapidement et qui englobe le cancer, les blessures, les maladies hépatiques, les troubles mentaux et les complications d’infections par le virus de l’immunodéficience humaine. Malgré des preuves incontestables démontrant que l’alcool est l’un des principaux facteurs de risque de décès prématuré et d’invalidité, rares sont ces pays qui intègrent des politiques globales de lutte contre l’alcoolisme dans leurs programmes de santé publique. Cet écart entre les preuves et les politiques est critique, car des interventions fondées sur des données scientifiques permettraient de réduire la charge de morbidité attribuable à l’alcool de près de 30% en l’espace d’une décennie. Nous avons mené une analyse des politiques afin de synthétiser les données provenant des pays à revenu faible et intermédiaire et de proposer un cadre global à plusieurs composants pour la lutte contre l’alcoolisme, inspiré de l’expérience acquise dans le domaine de la lutte contre le tabagisme. Les résultats documentés prouvent que l’intégration simultanée de la lutte contre l’alcoolisme dans le système de santé existant, la mise en place d’une réglementation et le renforcement des capacités institutionnelles permettent de réduire la consommation et de générer des retombées économiques notables. Cependant, l’ingérence de l’industrie de l’alcool dans l’élaboration des politiques, qui reflète l’approche historique du secteur du tabac, souligne la nécessité de mettre en place des mesures de protection explicites. Des recommandations fondées sur des données scientifiques concernant la mise en œuvre de politiques de lutte contre l’alcoolisme sont présentées à l’intention des agences internationales, des gouvernements nationaux, des systèmes de santé et de la société civile. Elles s’appuient également sur les enseignements tirés à l’échelle mondiale de la Convention-cadre de l’Organisation mondiale de la Santé pour la lutte antitabac. Actuellement, la convergence de données scientifiques solides, d’interventions éprouvées, de modèles réussis de mise en œuvre et d’un soutien politique croissant offre une occasion de faire progresser la lutte contre l’alcoolisme dans les pays à revenu faible ou intermédiaire. La question n’est pas de savoir si une lutte globale contre l’alcoolisme est réalisable dans des contextes aux ressources limitées, mais si la communauté sanitaire mondiale agira de manière décisive sur la base des données disponibles.

## Resumen

Los países de ingresos bajos y medios se enfrentan a una crisis de salud pública relacionada con el alcohol en rápida escalada, que abarca el cáncer, las lesiones, las enfermedades hepáticas, los trastornos de salud mental y las complicaciones de las infecciones por el virus de la inmunodeficiencia humana. A pesar de la evidencia concluyente de que el alcohol es un importante factor de riesgo de muerte prematura y discapacidad, pocos de estos países incluyen políticas integrales de control del alcohol en sus agendas de salud pública. Esta brecha entre la evidencia y las políticas es crítica, ya que las intervenciones basadas en la evidencia podrían reducir la carga de enfermedad atribuible al alcohol hasta en un 30% en una década. Se realizó un análisis de políticas para sintetizar la evidencia procedente de países de ingresos bajos y medios y proponer un marco integral multicomponente para el control del alcohol adaptado de la experiencia en el control del tabaco. Los logros documentados muestran que la integración simultánea del control del alcohol en el sistema sanitario existente, la introducción de regulación y el fortalecimiento de la capacidad institucional pueden reducir el consumo y generar importantes beneficios económicos. Sin embargo, la injerencia de la industria del alcohol en las políticas, que refleja el enfoque histórico de la industria del tabaco, pone de relieve la necesidad de protecciones explícitas. Se presentan recomendaciones basadas en la evidencia sobre la implementación de políticas de control del alcohol dirigidas a organismos internacionales, gobiernos nacionales, sistemas sanitarios y la sociedad civil. Estas recomendaciones también se basan en las enseñanzas globales derivadas del Convenio Marco de la Organización Mundial de la Salud para el Control del Tabaco. En la actualidad, la convergencia de evidencia científica sólida, intervenciones eficaces, modelos de implementación exitosos y un creciente apoyo político ofrece una oportunidad para avanzar en el control del alcohol en los países de ingresos bajos y medios. La cuestión no es si el control integral del alcohol es viable en contextos con recursos limitados, sino si la comunidad de salud mundial actuará de manera decidida sobre la base de la evidencia disponible.

## ملخص

تواجه الدول ذات الدخل المنخفض والدخل المتوسط، أزمة متصاعدة بسرعة في الصحة العامة مرتبطة بالكحول، تشمل السرطان والإصابات وأمراض الكبد والاضطرابات النفسية ومضاعفات عدوى فيروس نقص المناعة البشرية. ورغم وجود أدلة دامغة على أن الكحول عامل خطر رئيسي للوفاة المبكرة والإعاقة، فإن عدد قليل من هذه الدول تستعين بسياسات شاملة لمكافحة الكحول ضمن برامجها الصحية العامة. وهذه الفجوة بين الأدلة والسياسات تُعد بالغة الأهمية، لأن التدخلات القائمة على الأدلة يُمكن أن تحد من عبء الأمراض المترتبة على الكحول بنسبة تصل إلى 30% خلال عقد واحد. قمنا بإجراء تحليل للسياسات لتجميع الأدلة من الدول ذات الدخل المنخفض والدخل المتوسط، واقترحنا إطار عمل شامل متعدد المكونات لمكافحة الكحول، مأخوذًا من تجارب مكافحة التبغ. تشير الإنجازات الموثقة إلى أن دمج مكافحة الكحول بشكل متزامن في نظام الرعاية الصحية القائم، وطرح اللوائح، وبناء القدرات المؤسسية، يُمكنه أن يحد من الاستهلاك ويُحقق عوائد اقتصادية ملموسة. ومع ذلك، فإن تدخل صناعة الكحول في السياسات، والذي يماثل الأسلوب التاريخي لصناعة التبغ، يعزز الحاجة إلى عمليات حماية صريحة. تقدم التوصيات القائمة على الأدلة بخصوص تنفيذ سياسات مكافحة الكحول للهيئات الدولية، والحكومات الوطنية، وأنظمة الرعاية الصحية، والمجتمع المدني. كما تستند هذه التوصيات إلى الدروس العالمية المستفادة من اتفاقية إطار عمل منظمة الصحة العالمية حول مكافحة التبغ. إن تجميع الأدلة العلمية القوية والتدخلات المُثبتة، ونماذج التنفيذ الناجحة، والدعم السياسي المتزايد، يمنح حاليًا فرصة لتطوير مكافحة الكحول في الدول ذات الدخل المنخفض والدخل المتوسط. والسؤال ليس حول ما إذا كانت مكافحة الكحول الشاملة ممكنة في البيئات ذات الموارد المحدودة، ولكن حول ما إذا كان المجتمع الصحي العالمي سيتخذ إجراءً حاسمًا بناءً على الأدلة المتاحة.

## 摘要

中低收入国家正面临一场快速升级的酒精相关公共卫生危机，这场危机涉及癌症、意外伤害、肝脏疾病、心理健康障碍以及人类免疫缺陷病毒感染并发症。尽管有极具说服力的证据证明酒精是导致早逝和残疾的主要风险因素之一，但在这些国家中，仅极少数国家将综合性酒精控制政策纳入了公共卫生议程中。证据与政策之间的这一差距至关重要，因为循证干预措施能在十年内将酒精相关疾病负担降低高达 30%。我们开展了一项政策分析，旨在结合中低收入国家的证据并借鉴烟草控制经验，以提出一个综合性多元酒精控制框架。有据可查的成果表明，同时采取以下三项行动可有效降低酒精消费量并产生可观的经济收益：将酒精控制整合至现有卫生保健系统中、实施监管措施以及提升机构能力。然而，与烟草行业以往的做法如出一辙，酒精行业也在阻挠各项政策的实施，这凸显了构建明确保护措施的必要性。我们为国际机构、各国政府、卫生保健系统以及民间团体提供有关酒精控制政策实施的循证建议。此类建议还借鉴了《世卫组织烟草控制框架公约》中的全球经验教训。如今，中低收入国家可将以下四股力量凝聚起来以推动其酒精控制进程：强有力的科学证据、经验证切实可行的干预措施、成功实施模型以及日益增长的政治支持。问题的关键在于全球卫生共同体是否会基于可用证据果断采取行动，而非综合性酒精控制政策在资源有限地区是否切实可行。

## Резюме

Страны с низким и средним уровнем дохода сталкиваются с быстро нарастающим кризисом в сфере общественного здравоохранения, связанным с употреблением алкоголя. Этот кризис включает онкологические заболевания, травмы, болезни печени, психические расстройства и осложнения, связанные с ВИЧ-инфекцией. Несмотря на убедительные данные о том, что алкоголь является одним из ведущих факторов риска преждевременной смерти и инвалидности, лишь немногие из этих стран вводят комплексную политику борьбы против потребления алкоголя в свои программы общественного здравоохранения. Этот разрыв между доказательной базой и политикой имеет критическое значение, поскольку основанные на фактических данных меры вмешательства могли бы снизить бремя заболеваний, связанных с употреблением алкоголя, на 30% в течение десяти лет. Авторы проанализировали политику, чтобы обобщить данные из стран с низким и средним уровнем дохода и предложить комплексную многофакторную основу для регулирования потребления алкоголя, адаптированную на основе опыта борьбы против табака. Задокументированные достижения показывают, что одновременная интеграция мер по регулированию алкоголя в существующую систему здравоохранения, введение регламентирования и наращивание институционального потенциала могут снизить уровень потребления алкоголя и обеспечить значительную экономическую отдачу. Однако вмешательство алкогольной индустрии в формирование политики, повторяющее исторический подход к табачной индустрии, подчеркивает необходимость явных мер защиты. Основанные на фактических данных рекомендации по внедрению политики борьбы против потребления алкоголя представлены для международных организаций, национальных правительств, систем здравоохранения и гражданского общества. Эти рекомендации также опираются на глобальный опыт, накопленный в рамках Рамочной конвенции Всемирной организации здравоохранения по борьбе против табака. Сегодня сочетание убедительных научных данных, проверенных мер вмешательства, успешных моделей внедрения и растущей политической поддержки создает возможность для продвижения политики борьбы против потребления алкоголя в странах с низким и средним уровнем дохода. Вопрос заключается не в том, возможно ли внедрение комплексной политики борьбы против потребления алкоголя в условиях ограниченных ресурсов, а в том, будет ли мировое сообщество в сфере здравоохранения решительно действовать на основе имеющихся фактических данных.

## Introduction

Low- and middle-income countries are experiencing a surge in alcohol consumption that is contributing to a broad spectrum of health problems and producing substantial economic costs. The International Agency for Research on Cancer classifies alcohol as a group-1 carcinogen that has causal links to cancers of the oral cavity, pharynx, larynx, oesophagus, liver, colorectum and female breast.^1^ Globally, alcohol accounted for 741 300 new cancer cases in 2020, in a context where the incidence of alcohol-related cancer is 6.1% in men compared with 2.0% in women.^2^ Moreover, alcohol-attributable breast cancer resulted in the loss of 3.1 million disability-adjusted life years (DALYs) in 2021, with notable increases in disease burden in regions with a high proportion of low- and middle-income countries.^2^^,^^3^

Beyond cancer, alcohol also makes a substantial contribution to the burden of injuries, mental health conditions, domestic violence, infectious disease and cardiovascular disease. Global Burden of Disease data indicate that self-harm and interpersonal violence related to high alcohol use affected over 8 million people globally in 2021, with low- and middle-income countries disproportionately affected.^4^ In addition, alcohol is a major contributor to road traffic fatalities worldwide.^5^ The proportion was particularly high in studies from some countries: alcohol was implicated in 39% of fatal injuries in drivers in the United Republic of Tanzania,^6^ in 42% in Brazil,^7^ and in 25 to 45% in India.^8^ Alcohol also played a prominent role in 40 to 70% of cases of intimate-partner violence or assault presenting to health-care facilities, and pandemic-era studies in Mexico linked alcohol use to both the perpetration and experience of intimate violence, as well as to self-harm and suicidal ideation.^4^^,^^9^

Alcohol increases the burden of liver disease and is a leading cause of cirrhosis in settings where viral hepatitis is common.^10^ Moreover, the health impact of alcohol extends to cardiovascular disease: the prevalence of atrial fibrillation and hypertensive heart disease has risen among younger adults, and alcohol-attributable stroke accounted for 1.4 million DALYs in 2021.^11^^,^^12^ In addition, an estimated 40 to 70% of people with alcohol use disorders have comorbid mental health conditions, and alcohol consumption impairs the prevention and treatment of HIV infection by increasing the risk of transmission and reducing treatment adherence.^13^^–^^15^

The economic effects of alcohol consumption are substantial. Analyses across 12 low- and middle-income countries indicated that alcohol-attributable disease accounted for a substantial share of health budgets in 2009:^16^^,^^17^ the estimated proportion was 8.7% for cancer care, 12% for trauma care and 15% for mental health services. In severely affected low- and middle-income countries, total economic costs may reach 2 to 5% of gross domestic product.^16^^,^^17^ These multidomain health and economic effects underscore the need for comprehensive, cross-sectoral, policy responses to reduce alcohol consumption rather than siloed, disease-specific interventions.

## The policy–evidence disconnect

Despite the substantial health and economic burdens of alcohol consumption and strong evidence that interventions can be effective, comprehensive alcohol control policies are largely missing from the public health agendas of low- and middle-income countries. In 2024, only 56% of 124 reporting countries had a national alcohol policy in place, with the proportion dropping to around one quarter among low-income countries.^18^ In addition, only 38% of countries reported requiring warning labels on alcohol products, and more than half of 132 countries with excise taxes on alcohol failed to adjust them for inflation.^18^ Critically, alcohol advertising on the internet and social media, which is the fastest-growing marketing media, remains largely unregulated globally and comprehensive marketing bans and minimum pricing policies, both World Health Organization (WHO) SAFER best buys,^19^ have seen no meaningful progress since 2010 in most reporting countries.^18^ By contrast, many low- and middle-income countries benefited from WHO’s Framework Convention on Tobacco Control (FCTC),^20^ which spurred the systematic adoption of policies on tobacco control and yielded measurable health gains for their populations.

This gap between evidence on alcohol control and policy points to a major missed opportunity. The International Agency for Research on Cancer has shown that alcohol cessation rapidly reduces the activity of carcinogenic biological pathways, with health benefits observable within 6 to 12 months in population groups with high alcohol consumption.^1^ In addition, longitudinal studies report that alcohol-reduction interventions can produce a 15 to 30% decline in alcohol-related injury rates; a 20 to 40% reduction in interpersonal violence; and measurable improvements in mental health outcomes and HIV treatment adherence.^13^^,^^21^^,^^22^

Our policy analysis synthesized evidence on alcohol control policy from selected low- and middle-income countries with the aim of developing a comprehensive framework for alcohol control. We identified countries where national alcohol control policies had been successfully implemented. We selected these examples because: (i) they involved well-documented multicomponent programmes; (ii) robust evaluations had been conducted; (iii) the countries were geographically and culturally diverse; and (iv) implementation had been sustained for at least 3 years. These examples illustrate the feasibility of implementing alcohol control policies but do not necessarily represent best practice. We used data from the WHO Global Information System on Alcohol and Health,^23^ supplemented by policy documents and published evaluations, to assess the effectiveness of alcohol control policy across nine domains: (i) taxation and pricing; (ii) restrictions on availability; (iii) regulation of marketing; (iv) enforcement of a minimum legal drinking age; (v) drink–driving countermeasures; (vi) treatment services for alcohol misuse; (vii) alcohol screening and brief interventions; (viii) health information and education; and (ix) monitoring systems. We use the term policy dilution to describe the weakening of evidence-based provisions due to lobbying by the alcohol industry, political compromise or inadequate implementation mechanisms.^24^

## Industry tactics and informal production

The alcohol industry's targeting of low- and middle-income countries echoes the strategies used historically by the tobacco industry, as documented in internal corporate records and policy analyses.^25^^,^^26^ Economic development, rapid urbanization and aggressive marketing have led to increased consumption among population groups that previously abstained from alcohol, notably young people and women. Since 2010, alcohol marketing expenditure has risen substantially, and spending on digital and social media advertising, which is largely unregulated in most low- and middle-income countries, grew from 21% to an estimated 30% of the total alcohol advertising budget between 2019 and 2023 alone.^26^ Moreover, a study from Thailand found that alcohol advertising was pervasive despite regulatory limits, and that exposure to advertising was linked to an increased intention of adolescents to consume alcohol.^27^

Since 2015, sub-Saharan Africa has seen the establishment of over 150 new alcohol production facilities, which has created a policy conflict between economic development goals and the protection of public health.^25^ The alcohol industry tends to emphasize job creation and increasing tax revenues while downplaying health costs and other externalities. Moreover, corporate so-called responsible-drinking campaigns often counteract effective alcohol control policies and create a veneer of social responsibility for consumption. These tactics mirror those used historically by the tobacco industry.^24^

The existence of a large informal alcohol sector further complicates the implementation of alcohol control policies. In Uganda and the United Republic of Tanzania, unrecorded production accounted for roughly 60% of alcohol consumption in 2017.^25^ Moreover, it raised the risk of contamination and weakened regulatory oversight. Nevertheless, experience from different countries shows that comprehensive strategies to address both formal and informal alcohol markets can produce measurable benefits for public health.^28^^,^^29^

## Lessons from tobacco control

The success of WHO’s FCTC shows that ambitious global health goals are achievable with sustained political commitment, active civil society engagement and international cooperation. Low- and middle-income countries implemented comprehensive tobacco control measures, including taxation, smoke-free environments, prominent health warnings, marketing restrictions and smoking cessation services, and achieved measurable declines in the prevalence of smoking.^30^ A 2017 study which used WHO data from 126 countries that applied tobacco-control measures during the first decade of FCTC found that each additional demand-reduction measure, implemented at the highest level, was associated with an average relative decrease in smoking prevalence of 7.09%.^31^ Moreover, this association was consistent across United Nation subregions and World Bank income categories,^31^ which confirms the relevance of the findings to low- and middle-income countries.

Key factors in the Framework’s success that could be transferred to alcohol control include: (i) comprehensive, multicomponent strategies rather than isolated measures; (ii) sustained political commitment across electoral cycles; (iii) institutional safeguards to protect policies from industry interference; (iv) adequate resources for implementation and enforcement; (v) robust monitoring systems to enable evidence-informed policy adaptation; and (vi) integration with existing health systems rather than creating parallel structures.^30^^,^^32^

However, alcohol control presents distinct challenges that require an adapted approach, for example: (i) the existence of large informal production sectors; (ii) the deeper cultural integration of alcohol in some societies; (iii) a pattern of dual use, where moderate use coexists with harmful consumption; (iv) livelihoods dependent on the crops and production facilities associated with alcohol; and (v) the absence of a binding international framework comparable to the FCTC.^25^^,^^33^ Nevertheless, experience in low- and middle-income countries demonstrates that these challenges can be addressed through comprehensive, context-appropriate strategies.

## An alcohol control framework

Drawing on the lessons of tobacco control and on evidence from successful alcohol control programmes, we propose a comprehensive framework for alcohol control based on the simultaneous implementation of multiple components rather than sequential phases. The three principal components are: (i) integrating alcohol control into the existing health-care system; (ii) comprehensive regulatory policies; and (iii) building institutional capacity. Evidence from programmes in low- and middle-income countries shows that implementing these components simultaneously can maximize synergies and signal the government’s strong commitment to alcohol control. Examples from many countries demonstrate the feasibility and impact of this multicomponent approach.^28^^,^^29^^,^^33^

### Component 1

Alcohol screening and brief interventions can be rapidly integrated into routine health-care delivery across multiple platforms by building on the existing health infrastructure.^34^ For example, a pilot programme in the Indian State of Kerala that incorporated an alcohol risk assessment into an integrated noncommunicable disease risk assessment programme reached 2.3 million women and identified high-risk individuals at a substantially lower cost than a stand-alone programme.^35^

Comprehensive training of front-line health workers can build sustainable capacity for alcohol screening, brief interventions and referrals. Evidence from India showed that nurse-led brief interventions produced significant reductions in risky alcohol and tobacco use among older adults, with sustained effects observable at 3-month follow-up.^36^ In a randomized controlled trial (RCT) in Goa, India, lay counsellors trained in motivational, interview-based, brief psychological treatment (i.e. counselling for alcohol problems) achieved a remission rate of 54% among men with harmful drinking at 12-month follow-up compared with 32% among those receiving enhanced usual care alone.^37^ Further, economic analysis confirmed that counselling for alcohol problems produced better outcomes at a lower cost than usual care.^37^ In 2022, a systematic review of 75 RCTs across 13 low- and middle-income countries found that, despite consistent evidence that brief interventions and motivational interviewing are effective, alcohol treatment was hampered by a shortage of trained providers, limited integration into routine care and a lack of dedicated curriculum content for training programmes.^38^

Expanding treatment services remains critical. In most low- and middle-income countries, the treatment gap for alcohol-use disorders is substantial. For example, district-level data from the PRIME programme found that the 12-month contact coverage rate for probable alcohol use disorders in 2016 was as low as 2.8% in India, 5.1% in Nepal and 13.1% in Ethiopia.^39^ Structured psychosocial and pharmacotherapy programmes can achieve sustained abstinence rates of 15 to 35% at 12-month follow-up; and community-based, culturally adapted treatment models that incorporate peer support can improve engagement in settings where social stigma limits attendance at formal health-care facilities.^40^ Furthermore, implementation research from South Africa showed that integrated tuberculosis and alcohol treatment was feasible and acceptable in resource-constrained primary care.^41^

### Component 2

Regulatory interventions can deliver population-level effects that complement health-care measures. In the Russian Federation, a sequentially implemented, national alcohol control programme that included an increase in the excise tax on ethyl alcohol of 50%; the introduction of minimum unit pricing; a ban on night-time retail sales; and comprehensive marketing restrictions produced a 43% decline in per capita alcohol consumption between 2003 and 2016, accompanied by a 39% reduction in all-cause male mortality over the same period.^29^

Higher taxation and pricing consistently reduce consumption: typically a 10% price increase in low- and middle-income countries lowers consumption by 3 to 7%, with stronger effects observed among young people and low-income groups.^42^ A South African analysis showed that increased taxation both raised revenue and reduced consumption among price-sensitive population groups.^43^ Similarly, Thailand's progressive excise tax structure, which combined an *ad valorem* tax with specific taxes, achieved both its public health and revenue objectives.^44^ A modelling study in WHO’s European Region indicated that introducing a minimum alcohol tax share of 25% could avert more than 40 000 deaths annually, underscoring the population-level gains achievable through pricing policy.^45^ Moreover, a global analysis found that alcohol taxation could lead to substantial long-term health and economic benefits: over 50 years, a 20 or 50% increase in alcohol prices was estimated to generate a gain of 227 million or 547 million years of life, respectively.^46^ Around 44% of these health gains would occur in low- and middle-income countries, with low-income countries gaining the least because of lower baseline alcohol consumption.

Restricting alcohol availability by regulating trading hours can also reduce alcohol-related harm. For example, natural experiments in Brazil demonstrated that mandatory night closure of alcohol outlets reduced homicides by 10% and traffic injuries by up to 43%,^47^ which was consistent with a systematic review that confirmed the effectiveness of physical availability restrictions across low- and middle-income countries.^48^

Comprehensive bans on alcohol marketing that cover television, radio, outdoor advertising, sports sponsorship and digital platforms in the African Region have been reported to reduce both the likelihood that young people will start drinking and their overall consumption by an estimated 5 to 10%.^49^ Partial bans are less effective because the alcohol industry can respond by marketing through unrestricted communication channels. Thailand's comprehensive marketing restrictions offer a practical model,^27^ though ongoing monitoring is needed to detect circumvention via digital platforms and event sponsorship.

Drink–driving measures, such as lowering the blood alcohol content limit to 0.05% or 0.02%, random breath testing and licence suspensions, can reduce alcohol-related road crashes when enforcement is sufficiently frequent and visible.^50^

### Component 3

The Thai Health Promotion Foundation, called ThaiHealth, which is an autonomous body established in 2001 and funded by a 2% surcharge on both tobacco and alcohol excise taxes, coordinated policy implementation, supported evidence generation and engaged civil society to address harmful alcohol use and other risk factors for noncommunicable disease.^28^ Between 2005 and 2014, per capita annual alcohol consumption declined from 8.1 to 6.9 litres of pure alcohol – a reduction of approximately 15% over 9 years. This reduction could not be directly attributed to ThaiHealth alone given the parallel roles of taxation policy and regulatory agencies.^28^ Key enabling elements for ThaiHealth included dedicated and protected funding, multisectoral governance, transparent budget allocations and civil society engagement to safeguard ThaiHealth against industry interference.^28^

Safeguards against conflicts of interest are also important. The alcohol industry employs well-documented tactics to delay and dilute effective legislation, such as the manipulation of policy processes, litigation and the misuse of industry-sponsored research. Given the fundamental conflict of interest between commercial and public health objectives, governments must ensure that policy development and implementation are insulated from such interference and that parliamentarians are educated about industry tactics, as this will strengthen legislative support for comprehensive alcohol control measures.^51^

### Simultaneous implementation

Low- and middle-income countries with successful alcohol control programmes generally implemented these three components concurrently as far as resources allowed. In Thailand, ThaiHealth coordinated the generation of evidence, multisectoral campaigns and policy implementation simultaneously with the roll-out of regulatory measures.^28^ In Sri Lanka, community-based education programmes generated spontaneous community action groups that combined local advocacy for the closure of illicit alcohol outlets with lobbying of government ministries and civil society on broad policy.^52^ Table 1 summarizes our proposed evidence-based framework for alcohol control in low- and middle-income countries, accompanied by evidence of the successful implementation of each component and details of the key factors important for success.

**Table 1:** Evidence-based framework for alcohol control in low- and middle-income countries

Framework component	Core recommendations	Evidence from policy implementation in low- and middle-income countries	Key factors for success
Health-care system integration	(i) Integrate alcohol screening into primary care (e.g. into HIV services or maternal and child health programmes); (ii) train frontline health workers in brief counselling interventions; and (iii) establish referral pathways to community-based treatment services	(i) In Goa, India, a brief psychological treatment for harmful drinking (i.e. counselling for alcohol problems) implemented by trained lay counsellors led to remission in 54% of participants versus 32% in the usual-care group at 12-month follow-up, with counselling for alcohol problems producing better outcomes at a lower cost (i.e. it was the dominant strategy in a cost-effectiveness analysis);^37^ (ii) in a pilot RCT of a nurse-led brief intervention for risky alcohol and tobacco use among older adults in India, reductions in substance use risk scores were observed in both intervention and control arms but the between-group differences did not reach statistical significance, which highlights the need for adequately powered trials in this population;^36^ and (iii) in South Africa, an integrated tuberculosis and alcohol treatment programme was feasible and acceptable in resource-constrained, primary-care settings^41^	(i) Leverage existing health-care platforms to minimize cost; (ii) consider task-shifting to nurses and community health workers; (iii) build on proven training models rather than create new programmes; and (iv) integrate alcohol control with services patients already use
Regulatory policies	(i) Increase alcohol taxes to at least 75% of the total cost of alcohol; (ii) implement comprehensive marketing bans across all advertising platforms, including digital media; (iii) reduce alcohol outlet density and restrict trading hours; and (iv) enforce a drink–driving blood alcohol content limit of 0.05% or lower, with increased random roadside breath testing	(i) A global analysis revealed that a 20 or 50% alcohol price increase could generate 227 or 547 million years of life gained, respectively, over 50 years with 44% of gains occurring in low- and middle-income countries;^46^ (ii) in Thailand, progressive excise taxation and comprehensive marketing restrictions were associated with an approximately 15% decline in per capita annual alcohol consumption from 8.1 to 6.9 litres of pure alcohol between 2005 and 2014, though the reduction could not be directly attributed to any individual policy measure given concurrent regulatory and institutional factors;^28^ and (iii) in Russian Federation, a comprehensive programme that encompassed minimum unit pricing for vodka, escalating excise taxes on ethyl alcohol, a national ban on night-time retail sales and marketing restrictions was associated with a 43% decline in per capita alcohol consumption between 2003 and 2016, accompanied by a reduction in all-cause mortality of 39% in men and 36% in women over 2003–2018, with life-expectancy reaching a historic peak in 2018^29^	(i) Implement all alcohol control policies simultaneously within the first year rather than sequentially; (ii) use tax revenue to fund policy enforcement and health services; (iii) coordinate across government ministries (e.g. health, finance and law enforcement); and (iv) address both formal and informal alcohol production sectors
Institutional capacity and sustainability	(i) Establish an autonomous alcohol control committee with statutory authority and dedicated funding from alcohol tax revenues; (ii) implement conflict-of-interest policies prohibiting industry participation in policy development; and (iii) ensure the committee’s membership is multisectoral and includes participants from health care, law enforcement, education and civil society	(i) In Thailand, the Thai Health Promotion Foundation, an autonomous body established in 2001 and funded by a 2% surcharge on both tobacco and alcohol excise taxes, sustained multisectoral alcohol control coordination and evidence generation across successive governments. Per capita annual alcohol consumption declined from 8.1 to 6.9 litres of pure alcohol between 2005 and 2014, with dedicated protected funding, multisectoral governance and civil society engagement identified as key enabling elements;^28^ and (ii) systematic documentation of alcohol industry tactics, including the manipulation of policy processes, litigation and the misuse of industry-sponsored research, across multiple low- and middle-income countries underscores the need for explicit conflict-of-interest policies insulating government bodies and policy development processes from industry interference^51^	(i) Establish an alcohol control committee early in the programme to ensure its sustainability and to protect against political interference; (ii) ensure the alcohol control committee has a legislative mandate and multiyear funding so it is not dependent on annual budgets; (iii) ensure procedures are transparent to insulate the committee from industry influence; and (iv) establish a monitoring system to enable evidence-informed policy adaptation
Cultural considerations and community engagement	(i) Engage religious leaders and representatives of women's groups, youth organizations and community organizations to champion policy; (ii) frame alcohol control interventions around shared values (e.g. family welfare, child protection or financial stability); and (iii) adapt messaging and interventions to the local cultural and religious context	(i) In rural Sri Lanka, a community-based education programme involving street theatre, poster campaigns and group discussions was associated with significant, sustained reductions in hazardous drinking scores at 6 and 24 months, and with the spontaneous formation of a community action group that contributed to closure of illicit alcohol outlets. The programme's success was linked to culturally resonant messaging that emphasized family welfare and to strong community ownership;^52^ (ii) a scoping review of whole-of-community and intersectoral alcohol harm reduction interventions identified cultural appropriateness, collaborative community-driven engagement and the mobilization of existing community fora and action groups as consistent enablers of programme success in low- and middle-income countries, though robust comparative evidence of the superiority of multisectoral versus single-sector approaches remains limited in low- and middle-income settings;^53^ and (iii) in Malawi, an economic and relationship-strengthening intervention for couples with unhealthy alcohol use and HIV infections was associated with reduced interpersonal violence, improved relationship quality and perceived improvements in household finances^54^	(i) Build on existing community structures rather than create parallel systems; (ii) engage respected community leaders who can provide moral authority; (iii) combine culturally adapted messaging with evidence-based health information; and (iv) address alcohol use within a broader family and community well-being framework
Digital technology integration	(i) Ensure mobile screening platforms are integrated with national digital health identification systems; (ii) use artificial intelligence for predicting risk and targeting interventions; and (iii) ensure digital technologies are accessible for all, irrespective of literacy and the digital divide, while protecting privacy	(i) In Goa, India, a pilot RCT of an SMS-based brief intervention for hazardous drinking found that delivering the intervention through mobile phones was superior to an active control intervention in reducing alcohol consumption, thereby demonstrating its feasibility in a low-resource setting;^55^ (ii) in Kenya, a RCT of mobile phone-delivered motivational interviewing found that alcohol use scores at 1 month were significantly lower among participants who received the intervention than in those who did not, with effects sustained at 6 months;^56^ and (iii) a systematic review of digital interventions aimed at alcohol use and alcohol use disorders across low- and middle-income countries found they were broadly acceptable, feasible and effective in improving alcohol use outcomes, though evidence was limited by small sample sizes and short follow-ups and further evaluation is warranted before large-scale roll-out^57^	(i) Integrate mobile screening platforms with existing health information systems rather than use them alone; (ii) maintain human oversight of automated processes; (iii) use digital tools to complement, not substitute, face-to-face services; (iv) ensure data security and privacy protection; and (v) progressively scale up the use of digital tools according to their demonstrated effectiveness

HIV: human immunodeficiency virus; RCT: randomized controlled trial; SMS: short message service.

## Implementation challenges

### Cultural considerations

Alcohol control policies must take cultural sensitivities into account. In Sri Lanka, a community-based education programme involving street theatre, poster campaigns and group discussions in rural villages led to a sustained reduction in harmful alcohol use over 2 years compared with control villages.^52^ The programme's success was linked to culturally resonant messaging that emphasized family welfare, strong community ownership and social cohesion within a rural context.^52^

Research in low- and middle-income countries identified four key principles for implementing culturally appropriate programmes: (i) engage respected community leaders as champions; (ii) frame interventions around shared values (e.g. family welfare and child protection); (iii) adapt rather than import implementation methods; and (iv) build on existing community structures rather than create a parallel system.^52^ Systematic reviews of alcohol control interventions that involved the whole community or were intersectoral indicate that multistakeholder approaches outperform single-sector programmes.^58^

### Informal alcohol production

Tackling unrecorded alcohol production requires strategies that address both supply and demand. Effective measures include: (i) engaging the community to reduce the social acceptability of unregulated production; (ii) providing alternative livelihoods for producers; (iii) focusing enforcement on large-scale, illicit operations while supporting the transition of small producers into the legal market; (iv) enforcing quality standards and testing to mitigate the risk of contamination; and (v) designing taxes in a way that avoids incentivizing growth of the informal market.^25^

Experience shows that comprehensive policies can reduce both recorded and unrecorded consumption by shifting social norms and lowering overall demand.^29^ However, success depends on sustained enforcement and community mobilization rather than on regulatory measures alone.

### Digital technology

Digital technologies offer a scalable complement to policy interventions for alcohol screening and referral, particularly where specialist services are scarce. In India, a pilot RCT of a brief intervention for hazardous drinking based on short message service (SMS) text messages found that delivering the intervention through mobile phones was superior to an active control intervention in reducing alcohol consumption, thereby demonstrating its feasibility in a low-resource setting.^55^ In Kenya, an RCT of mobile phone-delivered motivational interviewing found that alcohol use scores at 1 month were significantly lower among participants who received the intervention compared with those who did not, with effects sustained at 6 months.^56^ Although digital interventions are broadly acceptable and feasible in low- and middle-income countries, evidence on their effectiveness is limited by small sample sizes and short follow-up periods and, consequently, further evaluation is needed before large-scale implementation.^57^

These digital tools should enhance, not replace, broader strategies. Key considerations for using digital technologies include: (i) ensuring they are accessible to all, irrespective of literacy and the digital divide; (ii) protecting privacy and data security; (iii) integrating digital tools with existing health information systems; (iv) maintaining human oversight of automated processes; and (v) using digital tools to complement, not substitute, face-to-face services.^57^

## Economic costs and returns on investment

Economic analyses in low- and middle-income countries indicate that alcohol-attributable disease consumes a disproportionate share of health budgets despite accounting for only a small share of overall caseloads.^16^^,^^17^ This burden reflects disease diagnosis at an advanced stage, the need for complex treatments, high recurrence rates and a substantial demand on social services.

A comprehensive alcohol control policy can yield a strong return on investment. In South Africa, higher alcohol taxation was associated with reduced health-care spending and a smaller productivity loss, thereby generating a net cost saving.^43^ At the household level, qualitative evidence from a combined economic and relationship-strengthening programme for couples with unhealthy alcohol use and HIV infections in Malawi, indicated that participants had improved family finances and livelihoods after reducing alcohol consumption. These findings demonstrate how reduced consumption can translate into tangible economic gains for vulnerable households.^54^

The WHO 2025 global investment case for noncommunicable diseases^59^ reports that implementing the full package of noncommunicable disease best buys, which included alcohol taxation, advertising bans and availability restrictions, could generate economic benefits worth seven United States dollars (US$) per dollar invested by 2035. Alcohol policy delivered the second-highest return among all interventions at US$ 9 per dollar invested.^59^ In addition, modelling studies indicate that substantial increases in alcohol excise taxes across low- and middle-income countries could generate hundreds of billions of dollars in additional annual revenue while simultaneously reducing consumption and associated health costs.^60^

## Conclusion

Experience with national alcohol control policies in the WHO South-East Asia Region shows that alcohol control is feasible in a diverse range of contexts and resulted in an 18 to 23% reduction in consumption and substantial economic benefits. Based on this experience, we propose an evidence-based framework for alcohol control in low- and middle-income countries (Table 1) and suggest actions that can be taken by different stakeholders (Box 1).

Box 1 **Actions for stakeholders, an evidence-based framework for alcohol control in low- and middle-income countries**

***Global public health organizations***

- Strengthen technical assistance by providing model legislation and policy implementation toolkits and establishing regional centres of excellence.
- Enhance WHO’s Global Information System on Alcohol and Health by expanding policy monitoring and impact evaluation.

***National governments***

- Implement comprehensive, multicomponent strategies simultaneously, including: (i) substantially increasing taxation; (ii) imposing comprehensive marketing bans; (iii) restricting alcohol outlet density; (iv) adopting zero-tolerance drink–driving policies supported by random roadside breath testing; and (v) making health warnings on packaging mandatory.
- Establish autonomous alcohol control committees that have statutory authority, receive dedicated funding from alcohol tax revenues and are protected from industry interference through conflict-of-interest policies.
- Integrate alcohol screening into primary health care (e.g. into maternal and child health, HIV, tuberculosis, mental health or noncommunicable disease services) and provide appropriate training for health workers.

***Health-care systems and professional organizations***

- Champion alcohol control as an important health goal through professional associations and medical societies.
- Implement systematic protocols for alcohol screening and brief interventions across all health-care settings, accompanied by ongoing training and quality assurance.
- Integrate content on alcohol harm into medical, paramedical and nursing education and training curricula.
- Document the burden of alcohol-attributable disease through facility-based surveillance to strengthen the evidence base.

***Civil society and community organizations***

- Build broad coalitions between members of health-care organizations, women's groups and youth organizations and religious leaders to create political pressure for action on alcohol control policy.
- Conduct evidence-based, advocacy campaigns to counter misinformation from the alcohol industry and highlight the health and economic impacts of alcohol consumption.
- Monitor the implementation of alcohol control policy and hold governments accountable.
- Support community-based alcohol misuse, prevention and treatment programmes adapted to the local context.

HIV: human immunodeficiency virus; WHO: World Health Organization.

Today, the convergence of definitive scientific evidence, proven interventions, successful implementation models and growing political will presents an unprecedented opportunity to advance alcohol control in low- and middle-income countries. Moreover, the success of WHO’s FCTC demonstrates that ambitious global health goals can be achieved with political commitment, civil society engagement and international cooperation. Alcohol control, which is facing industry tactics similar to those encountered with tobacco control, can draw on this established roadmap. However, making the best use of this opportunity requires a sustained commitment from stakeholders, adequate resources, explicit protection against industry interference and the political will to adopt comprehensive, evidence-based policies.

The evidence is clear, the interventions are proven and implementation models exist, the remaining question is whether the global health community will act decisively on this evidence.
